# Recording Visual Evoked Potentials and Auditory Evoked P300 at 9.4T Static Magnetic Field

**DOI:** 10.1371/journal.pone.0062915

**Published:** 2013-05-01

**Authors:** Jorge Arrubla, Irene Neuner, David Hahn, Frank Boers, N. Jon Shah

**Affiliations:** 1 Institute of Neuroscience and Medicine 4, INM 4, Forschungszentrum Jülich, Jülich, Germany; 2 Department of Psychiatry, Psychotherapy and Psychosomatics, RWTH Aachen University, Aachen, Germany; 3 JARA – BRAIN – Translational Medicine, Aachen, Germany; 4 Department of Neurology, RWTH Aachen University, Aachen, Germany; University Medical Center Groningen UMCG, The Netherlands

## Abstract

Simultaneous recording of electroencephalography (EEG) and functional magnetic resonance imaging (fMRI) has shown a number of advantages that make this multimodal technique superior to fMRI alone. The feasibility of recording EEG at ultra-high static magnetic field up to 9.4T was recently demonstrated and promises to be implemented soon in fMRI studies at ultra high magnetic fields. Recording visual evoked potentials are expected to be amongst the most simple for simultaneous EEG/fMRI at ultra-high magnetic field due to the easy assessment of the visual cortex. Auditory evoked P300 measurements are of interest since it is believed that they represent the earliest stage of cognitive processing. In this study, we investigate the feasibility of recording visual evoked potentials and auditory evoked P300 in a 9.4T static magnetic field. For this purpose, EEG data were recorded from 26 healthy volunteers inside a 9.4T MR scanner using a 32-channel MR compatible EEG system. Visual stimulation and auditory oddball paradigm were presented in order to elicit evoked related potentials (ERP). Recordings made outside the scanner were performed using the same stimuli and EEG system for comparison purposes. We were able to retrieve visual P100 and auditory P300 evoked potentials at 9.4T static magnetic field after correction of the ballistocardiogram artefact using independent component analysis. The latencies of the ERPs recorded at 9.4T were not different from those recorded at 0T. The amplitudes of ERPs were higher at 9.4T when compared to recordings at 0T. Nevertheless, it seems that the increased amplitudes of the ERPs are due to the effect of the ultra-high field on the EEG recording system rather than alteration in the intrinsic processes that generate the electrophysiological responses.

## Introduction

Simultaneous recording of electroencephalography (EEG) and functional magnetic resonance imaging (fMRI) has shown a number of advantages that make this multimodal technique superior to fMRI alone [1]–[9]. The possibility of integrating the trial information provided by EEG into fMRI analyses can enrich the results and add new perspectives on brain function. This is particularly important in studies where variability in responses may be indicative of stimulus processing modulation [10]–[12]. Electrophysiological data complements the high spatial resolution information obtained with MR at ultra high fields (≥7 T) with high temporal resolution information of brain function. Simultaneously acquired EEG/fMRI has already been shown to be useful in the detection of epileptogenic lesions and treatment of medication resistant epilepsy [13], [14]. The benefits of simultaneous EEG/fMRI at ultra-high fields would contribute to many aspects of cognitive neuroscience, resting state studies, pharmacological studies, sleep studies or evoked potential studies [4], [15], [16].

The major strengths of EEG are the high temporal resolution, non-invasiveness and relatively straightforward preparation. An important disadvantage of the combination, however, is the highly contaminated signal when EEG is acquired inside an MR scanner. In the first place, gradient artefacts occur due to electromotive forces (EMF) induced in the EEG leads by the switching magnetic fields used in fMRI. However, due to the consistency of this artefact over time, the subtraction methods, such as the average artefact template, are quite successful [17]. The ballistocardiogram (BCG) artefact, produced by cardiac pulse-related movement of the scalp electrodes inside the magnetic field, constitutes a bigger challenge. The exact source of pulse artefact is unclear but it is related to a number of factors including pulsatile blood movement, and head movements. Movement of electrically conductive material in a magnetic field results in an EMF; the motion related to cardiac activity can lead to EMF in the circuit between the subject and the recording equipment, which results in measured voltages and artefacts in the recording. The so-called “Hall effect” adds further pulse-related artefacts to the recordings; a small voltage is induced by fluctuations in blood flow velocity in nearness to the electrodes [18]. Thus, a number of methods for correction of the BCG artefact exist. Independent component analysis (ICA), for example, assumes stationarity of the sources and, due to the variability of the BCG artefact, shows a considerable spatial variation across its occurrence, making the correction problematic. Based on the assumption that there is no dependency between the sources of spontaneous EEG and the pulsatile blood-flow, the first ICA-based methods for correction of the BCG artefact were based on manual selection of the artefactual components [19]. Optimal basis set (OBS) [20], on the other hand, is based on the assumption that each BCG artefact occurrence in each channel is independent of any previous occurrence. OBS captures the variations of the BCG artefact along the time axis and applies principal component analysis to all occurrences per channel.

Removal of the BCG artefact in simultaneous EEG/fMRI studies performed in magnetic field strengths between 1.5 and 3 Tesla appears to be a solved problem using OBS prior to ICA, as suggested by Debener and colleagues, instead of applying ICA directly on the EEG data [10].

With the development and implementation of human MR scanners with magnetic fields of 7T and above, the BCG artefact removal problem must be revisited. In a previous study, Neuner and colleagues demonstrated, from a technical point of view, that electrophysiological recordings in 9.4T static magnetic fields are feasible [21]. From the data quality point of view, they showed that the BCG artefact was not reduced sufficiently by automatic methods such as OBS and led to sub-optimal correction.

The results of Neuner and colleagues also showed that the speed of sensory perception was not altered by the 9.4T magnetic field. Nevertheless the speed of cognitive functions was not evaluated, which leaves the question open to investigate the effect of 9.4T magnetic field to this regard. Recent research in this field has shown that ultra-high fields do not seem to have any persisting influence on the attention networks of human cognition immediately after exposure [22]. Nevertheless the focus of such investigations have been the performance of neuro-cognitive testing before and after the MRI examinations. Recent evidence shows that static magnetic fields as high as 7.0 T did not have a significant effect on cognition [23].

The evoked P300 is a positive peak that becomes evident approximately between 250 and 500 ms after a stimulus [24]. It appears commonly during an ‘oddball paradigm’, in which frequent and target stimuli are presented. The amplitude of the P300 increases with lower probability and higher discriminability of targets and it is believed to be a neural signature of attention and working memory required for an appropriate response to environment stimuli [25].

After demonstrating the feasibility of EEG-recordings in a 9.4T static magnetic field, and motivated by the future applications of EEG at ultra-high magnetic fields, we have designed a study to answer the following questions:

- Is it possible to record visual evoked potentials (VEP) in a 9.4T static magnetic field, since fMRI studies including visual stimulation could be applied in future?
- Is it possible to record auditory P300 in a 9.4T static magnetic field? And if this were possible, are the very early stages of cognitive processing altered by the ultra-high magnetic field?
- Do ICA-based methods for BCG artefact correction sufficiently reduce the artefacts so that ERPs could be identified?

## Materials and Methods

EEG data were recorded from 26 healthy volunteers (7 female) with a mean age of 34.08 (SD 12.76) years. Written informed consent was obtained from all subjects and the study was approved by the Ethics Committee of the Medicine Faculty of the Rheinisch-Westfälischen Technischen Hochschule Aachen (RWTH Aachen University). The study was conducted according to the Declaration of Helsinki. EEG data were recorded using Brain Vision Recorder (Brain Products, Gilching, Germany) and a 32-channel MR compatible EEG system (Brain Products, Gilching, Germany). The EEG cap (BrainCap MR, EasyCap GmbH, Breitbrunn, Germany) consisted of 31 scalp electrodes distributed according to the 10–20 system and one additional electrode for recording the electrocardiogram (ECG). Data were recorded relative to an Fpz reference and a ground electrode was located at AFz (10-5 electrode system) [26]. Data were sampled at 5000 Hz, with a bandpass of 0.016–250 Hz. Impedance at all recording electrodes was kept below 10 kΩ. During data acquisition the helium pump of the 9.4T MR system was kept running. Data recorded with and without the helium pump active (not presented) showed that the compressor did not contribute significantly to noise levels.

### EEG Recordings

EEG data were recorded from each subject outside of the scanner (0T) and inside a Siemens 9.4T human, whole-body MR scanner (Siemens Medical Systems, Erlangen, Germany). Measurements outside the scanner were performed with the subject in a supine position to be consistent with measurements made inside the scanner. Auditory and visual stimuli were presented separately and recorded at 9.4T and 0T. For the 9.4T recordings the scanner described above was used and subjects were positioned at the isocentre of the scanner. The 0T measurements were performed in a mock scanner where the scanner environment can be realistically created without the effects of the static field on the EEG signal.

Auditory stimuli were delivered via headphones using Presentation software (Version: 11.0, Neurobehavioral Systems. Albany, California) and an external driver unit for earphones (model SRM-252II. STAX ltd. Saitama, Japan). Extension cables made of copper were used between the driver unit and the headphones (model SRE-750. STAX ltd. Saitama, Japan). In the auditory oddball task, subjects were presented with a series of high (1000 Hz) “task relevant” target tones and lower (500 Hz) “task irrelevant” tones of 50 ms duration, rise and fall time, 5 ms, 85 dB, and inter-stimulus interval (ISI) between 2 and 14 s. Target probability was 20%. There was a delay of 26 ms between the stimuli marker in the EEG recording and the actual presenting of the tones to the volunteers. The delay between the EEG marker and the tones was measured using an oscilloscope by establishing the time between the marker signal and the onset of the tones. This time was constant and was due to processing times in the sound card of the stimulation computer. Subjects were instructed to press the button with the index finger of the right hand at each target tone.

Visual stimuli were delivered via two optic fibres using Cambridge Research System and light-generator box. The optic fibres were adapted to custom-made goggles placed over the subject’s field of vision. The stimuli consisted in 200 flashes of white light with an intensity of 12 cd/m^2,^ duration of 500 ms and ISI between 2–4 s. There was no significant delay (<1 ms) between the digital marker and the onset of the light pulse detected in the visual stimulation.

### EEG Data Processing

EEG data were first processed in Brain Vision Analyzer (Version 2.02, Brain Products, Gilching, Germany) where the following steps were performed: down-sampling to a rate of 250 Hz, filtering at 0.16–20 Hz with a notch filter at 45–55 Hz and re-referencing to the average in case of auditory stimulation data and to Fz in case of visual stimulation according to the ISCEV (International Society for Clinical Electrophysiology of Vision) standard for clinical visual evoked potentials [27]. The data recorded in the 9.4T static field were corrected for BCG artefacts by means of ICA, where the components were visually inspected and those whose activity was related to the heartbeat events in the ECG signal were excluded. In order to obtain independent components (IC) a restricted Infomax ICA algorithm was applied to the whole data, including 512 steps and automatic determining of the components with eigenvalue trigger of 0.001. All data were analyzed by the same trained operator. The data recorded at 0T and 9.4T were exported to EEGLAB (http://sccn.ucsd.edu/eeglab/) [28] for further analysis. Data were then segmented around the event markers, 50 ms before and 250 ms after the stimulus in case of visual paradigm and 50 ms before and 450 ms after the stimulus in case of target auditory stimuli. Segment rejection was performed by visual inspection and recognition of muscle and high amplitude artefacts. The presence of evoked potentials was first evaluated at Pz channel for the auditory oddball paradigm in search of P300, and at Oz for visual paradigm in search of P100. The values of amplitude and latency were exported for statistical analysis.

The segmented data were later subjected to extended infomax ICA [29] using the runica algorithm [30] from the EEGLAB toolbox. The resulting ICs were inspected for topography, ERP signal and consistency across single trials to determine event related potential components.

The topographic criterion for choosing components related to the VEP was a distribution of signals arising in the occipital lobes. The shape of the signal was then evaluated at the Oz channel, where clear positive signals with latency between 75 and 175 ms were sought (P100). In this regard, the data recorded at 0T in channel level served as example for latency and shape when choosing the components. The chosen components were kept while the rest were excluded. Amplitude and latency values of the resulting signals at Oz were exported for statistical analysis.

The same procedure was applied for the data recorded during auditory stimulation. The topographic criterion was positive signal arising in the middle-parietal area, the shape of the signal was evaluated in Pz in search of positive peak between 275 and 400 ms. Data recorded at 0T served as examples again. The chosen components were kept and the rest were excluded; amplitudes and latencies at Pz were exported for statistical analysis.

All data were analyzed first by one operator, the chosen/rejected ICA components were discussed with a second operator and only when both operators agreed, a component was chosen or rejected.

## Results

### Visual Stimulation

**Figure 1:**
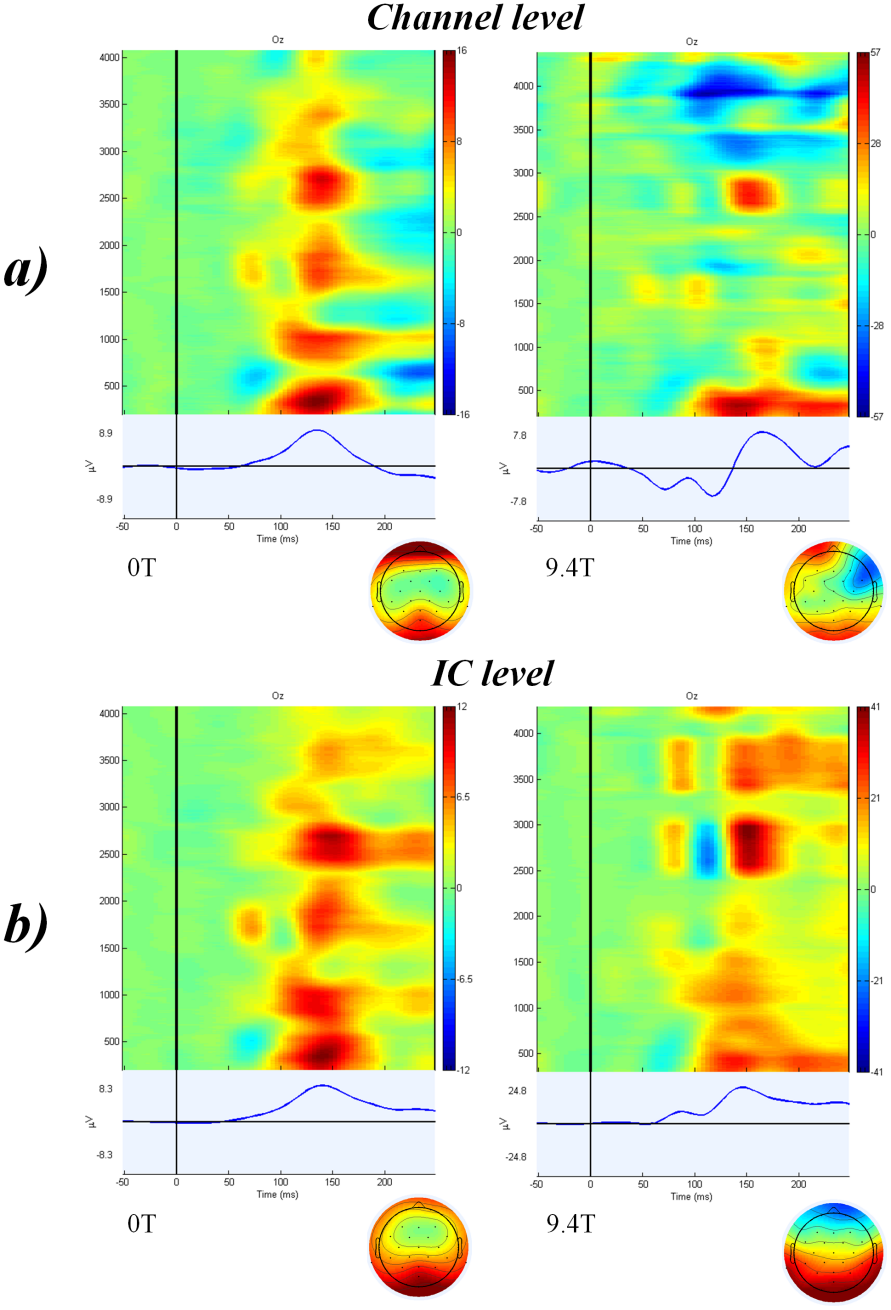
Average of all trials in 26 subjects after visual stimulation at (a) channel level after ICA-based BCG artefact correction and (b) IC level. Figures on the left correspond to data recorded at 0T and figures on the righ to data recorded at 9.4T.

**Figure 2:**
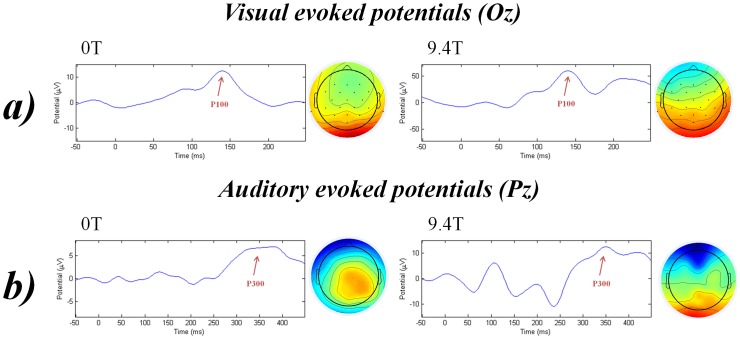
Example of (a) visual evoked P100 and (b) auditory P300 (target stimuli) at 0T and 9.4T at IC level in one representative subject.

Clear VEPs in 24 subjects were identified in the individual channel data at 0T but only traces of ERPs were found in 5 subjects at 9.4T static magnetic fields. Average of all trials in 26 subjects at channel level of data recorded at 0T and 9.4T can be seen in Figure 1-a. Independent components representing clear VEPs were found in all 26 subjects at 0T. It was possible to identify clear independent components representing VEPs from 21 subjects in data recorded at 9.4T. Average of all trials in 26 subjects at IC level of data recorded at 0T and 9.4T can be seen in Figure 1-b. Data from one representative subject can be seen in Figure 2-a. A paired t-test showed no significant difference in the latencies of the visual P100 recorded at 0T (M = 138.15 ms, SD = 13.54) and at 9.4T (M = 141.07 ms, SD = 16.18); t(25) = −0.842, p = 0.408. There was a significant difference in the amplitude of the visual P100 recorded at 0T (M = 7.49 µV, SD = 4.95) and at 9.4T (M = 23.69 µV, SD = 27.13) static magnetic fields; t(25) = −3.334, p = 0.003.

### Auditory Oddball Paradigm

**Figure 3:**
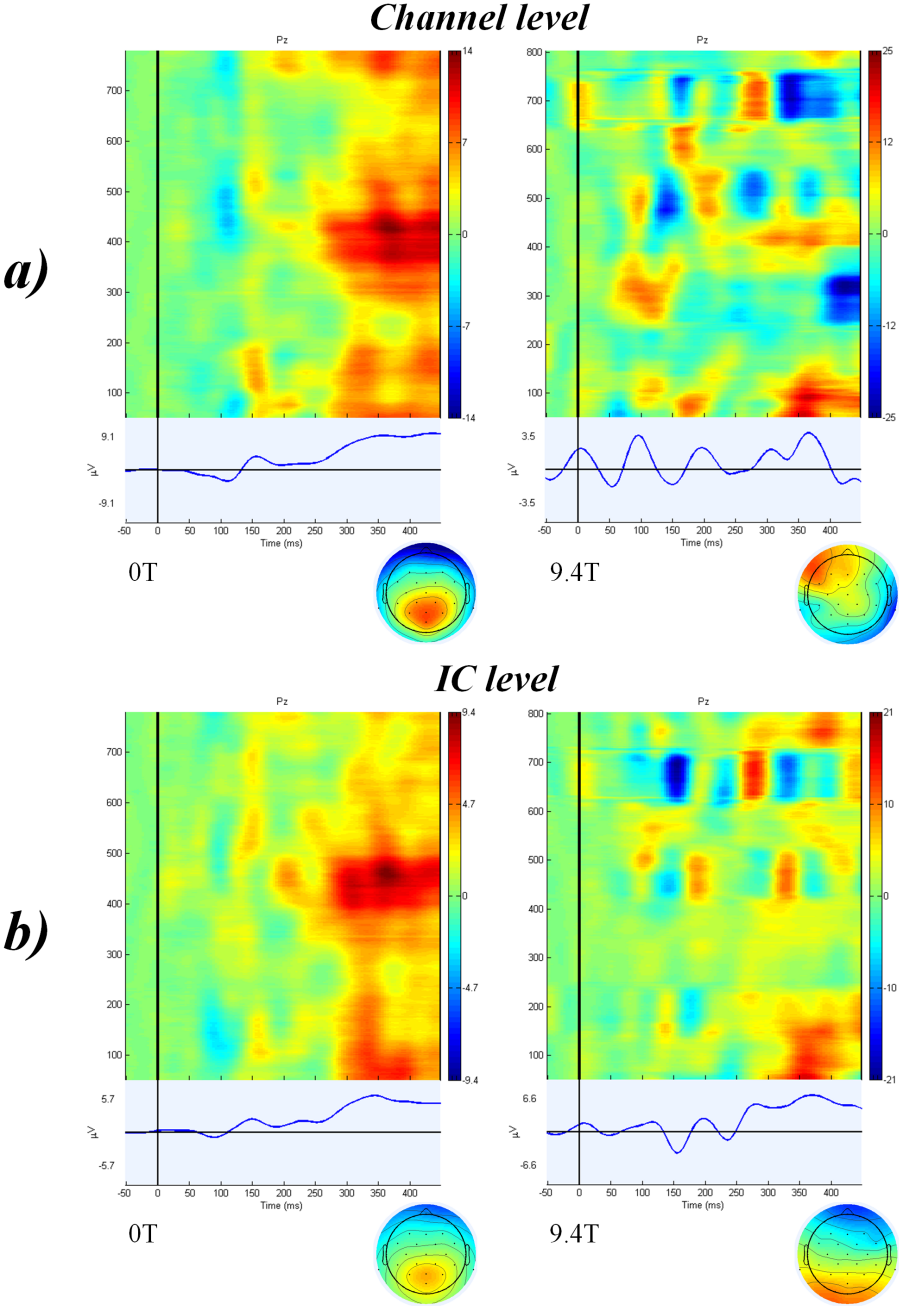
Average of all trials in 26 subjects after auditory stimulation (target stimuli) at (a) channel level after ICA-based BCG artefact correction and (b) IC level. Figures on the left correspond to data recorded at 0T and figures on the righ to data recorded at 9.4T.

Clear auditory P300 peaks were identified in 25 out of 26 subjects in individual channel data at 0T but only traces of ERPs were found in 14 subjects at 9.4T. Average of all trials in 26 subjects at channel level of data recorded at 0T and 9.4T can be seen in Figure 3-a. Independent components representing clear P300 peaks were found in all subjects at 0T. It was possible to identify clear independent components representing auditory P300 from 19 subjects in data recorded at 9.4T. Average of all trials in 26 subjects at IC level of data recorded at 0T and 9.4T can be seen in Figure 3-b. Data from one representative subject can be seen in Figure 2-b. Paired t-test showed no significant difference in the latencies of the auditory P300 recorded at 0T (M = 353.23 ms, SD = 27.7) and at 9.4T (M = 348.61 ms, SD = 33.63); t(25) = 0.519, p = 0.608. There was a significant difference in the amplitude of the auditory P300 recorded at 0T (M = 5.15 µV, SD = 2.37) and at 9.4T (M = 9.76 µV, SD = 7.57); t(25) = −3.128, p = 0.004.

## Discussion

We performed a study to investigate the possibility of recording VEP and auditory evoked P300 inside a 9.4T static magnetic field, specifically to determine whether meaningful EEG information could be recovered from the data after removal of the BCG artefact by means of ICA. We also investigated whether the early stages of cognitive processing are altered by the ultra-high magnetic field of 9.4T. The results of this study confirm the feasibility of recording evoked potentials at 9.4T static magnetic fields [21] and extend the possibilities to visual P100 and auditory P300 ERPs. The ICA demonstrated to be effective in removal of the BCG artefacts, and also for identifying the ERPs. It was not possible to find clear ERPs in all cases when EEG was recorded at 9.4T in individual channel data directly after removing the BCG artefact. We believe this effect is due to artefacts generated in the electrodes and cables of the EEG system when they move within the magnetic field.

Our results show that the latencies of the ERPs were not different when the stimulation was performed at 0T or 9.4T static magnetic field strengths. This finding supports the assumption that the speed of primary sensory perceptions and early cogntivie processing are not altered by the 9.4T static magnetic field. The amplitudes of the ERPs were higher when EEG was recorded at 9.4T in comparison to recordings at 0T. Even though this difference was statistically significant, it seems that the increased amplitudes of the ERPs are due to the effect of the ultra-high magnetic field on the amplifier rather than alteration in the intrinsic processes that generate the cortical responses. The latter is also supported by the topographic distribution of the average signals across the subjects, where the topographic origins of the signals are preserved in the 9.4T recordings (Topographic view in Figure 2). Though the EEG amplifier we used was MR compatible, shielded and specially designed for MR experiments, the 9.4T magnetic field seemed to overcome the amplifier capabilities and subsequently affected all the signals conducted in the device. This effect needs further investigation.

ICA is a highly valuable technique in which each component is specified by a fixed linear spatial filter that determines a time course of activation during each response condition, plus a fixed pattern of strengths at each of the scalp electrodes. Data from N electrodes can be reconstructed as the sum of the N independent components. Averaged ERPs are thought to be caused by synchronous activity in pyramidal cells in the activated areas. Because volume conduction through the cerebrospinal fluid, skull, and scalp is thought to be linear, sensory ERPs are assumed to sum stable potentials associated with activation in each stimulated area [30]. Thus, ICA is able to decompose the signal according to their different sources and allows the identification of the sources of the ERPs and permits the reconstruction of the signal up to 9.4T static magnetic fields. In the study of Neuner and colleagues, even though OBS was used to correct the BCG artefacts, ICA was necessary to identify components representing ERPs. In this study we have used ICA for correction of the BCG artefacts and found the same problem, which lead us to conclude that standard correction methods are not sufficiently effective for cleaning EEG data recorded at 9.4T. This is particularly evident in the analysis of data recorded at 9.4T, where only few components represented stimuli locked events after ICA, and the rest were related either to artefacts or background signal. Deeper investigation on the nature of artefacts in recordings at 9.4T is needed to develop appropriate correction tools.

Recording electrophysiological data in ultra-high fields up to 9.4T is a promising field that is also bringing numerous challenges. When fMRI/EEG investigations start further challenges will arise from the additional use of magnetic field gradients for image encoding, such as gradient-related artefacts. These results need to be replicated within a simultaneous fMRI experiment to determine the effects of gradient artefact removal on data quality at ultra high fields.

### Conclusions

We were able to retrieve visual P100 and auditory P300 evoked potentials from data acquired in a 9.4T static magnetic field after correction of the BCG artefact and reconstruction of the ERP signal using ICA. The latencies of the ERPs recorded at 9.4T were not different from those recorded at 0T suggesting that the speed of primary sensory perceptions and early cognitive processing are not altered by the 9.4T static magnetic field. The amplitudes of the ERPs were higher at 9.4T when compared to recordings at 0T. We believe this is due to the effect of the ultra-high field on the EEG recording system rather than alteration in the intrinsic processes that generate the electrophysiological responses; further work is required to estabish the veracity of this assertion.
